# Valorization of Two African Typical Crops, Sorghum and Cassava, by the Production of Different Dry Pasta Formulations

**DOI:** 10.3390/plants12152867

**Published:** 2023-08-04

**Authors:** Elena Galassi, Laura Gazza, Francesca Nocente, Phabiola Kouagang Tchakoutio, Chiara Natale, Federica Taddei

**Affiliations:** CREA Research Centre for Engineering and Agro-Food Processing, Via Manziana 30, 00189 Rome, Italy; elena.galassi@crea.gov.it (E.G.); laura.gazza@crea.gov.it (L.G.); francesca.nocente@crea.gov.it (F.N.); ktphabiola@yahoo.fr (P.K.T.); chiara.natale@crea.gov.it (C.N.)

**Keywords:** pasta, cassava, food-grade sorghum, dietary fiber

## Abstract

Mediterranean diet is changing to keep up with the increasingly multiethnic Italian society. With food being considered as a means of integration, innovative foods capable of mixing different raw materials could be of interest. In this work, some of the most consumed African foods such as sorghum, cassava, and durum wheat were used to produce wholegrain spaghetti to valorize their nutritional and sensorial aspects and to combine Italian and foreign tastes. Different pasta formulations (cassava, semolina, cassava:semolina, cassava:sorghum, cassava:durum wheat whole meal, sorghum:semolina) were developed and compared for their content of proteins, total starch, resistant starch, amylose, fiber, total antioxidant capacity, ash, cooking quality and sensorial characteristics. The enrichment of cassava flour with durum wheat and sorghum wholegrain enhanced the total antioxidant capacity, protein, and fiber content with respect to 100% cassava pasta. The presence of cassava or sorghum resulted in a high diameter variability of pasta samples, lower water absorption, and shorter optimal cooking time with respect to semolina pasta. Sensory evaluation of cooked pasta revealed better scores in blends containing semolina. Although the obtained pasta samples were interesting for their nutritional aspects, further adjustments are required in the pasta-making process to improve pasta quality.

## 1. Introduction

In 2022, there were nearly eleven million African citizens in Europe according to the Africa Center for Strategic Studies [1]. Currently, there are almost 5 million foreign expats in Italy, representing about 8.5% of the national population, and among those, 22.5% come from Africa [2]. Foreign people often adjust their food habits to a new cultural environment following the acculturation process, but also because of difficulties in traditional food availability. Also, the Mediterranean diet is changing to keep up with the increasingly multiethnic Italian society, and in this regard, the Italian Pediatric Society has even created a transcultural food pyramid and introduced foods that are new to Italians, like millet, sorghum, amaranth, quinoa, mango, and cassava [3]. However, with food being considered as a possible means of acculturation and integration, continuous research on different raw materials and new processing technologies to obtain innovative foodstuffs that meet Italian and foreign tastes is of remarkable interest.

Among the most consumed African foods, durum wheat, sorghum, and cassava represent the major source of carbohydrates in Africa; the inclusion of these crops on the national scene would be both a sustainable choice, suitable for facing climate change, and an opportunity for the enhancement of cultural inclusion and the development of new healthy food products. Durum wheat (*Triticum durum* Desf.) is a significant food crop in the world, with an estimated 33.8 million tons of annual global production in 2021 [4]. The nutritional content of durum wheat grain consists, on average, of 13% proteins, 2.9% lipids, 53.9% starch, 9.8% total dietary fiber [5]. The annual production of the main durum wheat food product, pasta, was estimated at 16.9 million tons in 2021. On a global scale, most of its consumption and production is in Europe, South America, and the United States, whereas the African continent accounts for only for 13.8% of total pasta production [6].

Sorghum (*Sorghum bicolor* Moench) is the fifth most widely grown cereal crop after wheat, rice, corn, and barley. Africa is the main producer (16.5 million tons), followed by United States, Mexico, and India; EU countries produce only 750,000 tons per year, while 60 million tons are harvested each year worldwide [7]. Sorghum can be a potential crop for meeting food security and climate change challenges, being able to adapt to shortage of water and warmer temperatures. In this context, it could constitute promising raw material for enhancing diversification in nutrition and sustainable agriculture. Sorghum grain is essentially made up of carbohydrates (70–80%), proteins (8–18%), lipids (1–5%), and dietary fiber (19%) [8]. Moreover, sorghum shows a great concentration of bioactive compounds and good technological properties that could contribute to the development of healthy gluten-free foods [9]. Sorghum is generally consumed whole grain or processed into flour, from which traditional meals are prepared such as bread, porridge, boiled grains, and fried products. Cassava (*Manihot esculenta* Crantz) is the primary food source for more than 800 million people, and in Africa, it is the fourth most important crop commodity [10], with an average consumption of 50 kg/capita/year [11] and represents the third largest source of carbohydrates in the Tropic countries [12]. Indeed, cassava roots are a good source of energy, being composed almost exclusively of carbohydrate from 80 to 90% [13]; conversely, it is low in protein (1.5–3.5%) and lipids (0.5–1.5%) and contains 1.4–8.5% of dietary fiber [14]. The global cassava production stands slightly over 11 tons/ha and over 63% of the 303 million tons produced globally in 2019 was from Africa [15].

Cassava is adaptable to low fertile lands and to less favorable climatic conditions such as drought [16]. The edible parts of this crop are the roots, rich in starch and poor in protein. Postharvest physiological deterioration in cassava roots reduces shelf life and makes its distribution laborious [17]. Cassava is basically processed into fermented (e.g., cassava bread, fermented cassava flour, fermented starch) and unfermented products (e.g., tapioca, cassava chips and pellets, unfermented cassava flour and starch). New food uses for cassava include as flour in gluten-free or gluten-reduced products (e.g., bread, biscuits, etc.) [18].

Dry pasta-specific attributes in terms of versatility, low cost, long shelf life, easy preparation, worldwide diffusion make it the optimal matrix for enrichment with functional molecules [19] or other kinds of non-wheat flours and ingredients. Indeed, pasta has been the object of many supplementation strategies to improve its nutritional and health potential [20]. Different attempts to produce sorghum and cassava pasta were conducted providing high value-added ingredients by improving nutritional, technological, and functional properties [21,22,23].

In this work, sorghum, cassava, and durum wheat flours were combined in different formulations to produce a novel pasta in order to valorize these crops by the production of spaghetti, one of the most popular Italian dishes consumed in the world, to meet the tastes of different cultures. The nutritional and cooking properties of the different pasta blends were evaluated and compared to semolina spaghetti.

## 2. Results and Discussion

### 2.1. Chemical Characterization and Total Antioxidant Capacity of the Raw Materials

The content in protein, amylose, dietary fiber (TDF), ash, and antioxidant capacity (TAC) were found in ascending order in cassava flour (CF), sorghum (SF), semolina, and durum wheat (DWF) whole meal flours, whereas total starch (TS) was significantly (*p* ≤ 0.05) higher in cassava than in DS, SF, DWF in that order (Table 1).

The protein content of cassava flour was nearly six- and fourfold less than durum wheat and sorghum whole meal flours, respectively. The highest starch value was detected in CF, about 36% and 13% more than those in DWF and SF, respectively. The high starch content makes cassava a valuable food source of carbohydrates and a raw material for extracting starch useful in food and nonfood industrial applications. Resistant starch (RS) content in all raw materials was found below quantification limit of the method; however, the highest content was observed in sorghum whole meal and the lowest in semolina. CF showed the lowest value also in amylose content, confirming the positive relation existing between resistant starch and amylose content, as previously observed by [24]. As expected, total dietary fiber values revealed a significantly (*p* ≤ 0.05) higher content in cereal whole meal flours (almost +70% on average) than in cassava flour and semolina (Table 1). The same tendency was observed for antioxidant activity, significantly higher in durum wheat (+200%) and sorghum and semolina (+81%) whole meal flours than in CF (Table 1). Indeed, the presence of antioxidants, mostly located in the outer layers of cereal kernels, contributed most to the high TAC values in whole meal flours [25]. As observed for TAC, the whole meal flours showed values of ash content more than twice with respect to cassava. The low TAC level, protein, and TDF content of cassava flour suggests the opportunity to obtain enrichment with other raw materials to obtain products with satisfying nutritional potential, as indicated by Lawal et al. [22,26], who found a significant increase in TAC, proteins, and fiber values in cassava flours when enriched with amaranth and pumpkin.

### 2.2. Chemical Characterization and Total Antioxidant Capacity of Dry Pasta

Preliminary tests were conducted in order to select the best pasta formulations, spaghetti shape in terms of technological parameters, and pasta-making aptitude. The selected formulations are described in the Materials and Methods section. Durum wheat semolina pasta (DS100) showed the highest protein content, whereas cassava pasta (C100) reported the lowest value, followed by spaghetti obtained from the blend of cassava and semolina (C50:DS50) and cassava and sorghum flour (C50:S50) (Table 2). The presence of cassava represented the main responsible for the low protein content of pasta formulations; however, when cassava flour was combined with durum whole meal wheat (C50:DW50), the protein content increased, on average, by at least fivefold compared to that found in spaghetti made only of cassava flour (C100). Contrastingly, the replacement of cassava with 50% of durum wheat semolina or sorghum whole meal flour led to a smaller increase, almost 2.5-fold, in protein content. The contribution of external layers on the protein content of whole meal products is well known [27]. The significant increase in protein content observed when cassava was combined with sorghum or durum wheat (whole meal or semolina) highlight how blending flours is a valid strategy for overcoming the low protein content that limits the nutritional potential of cassava. As already observed in cassava flour, total starch content was higher in cassava spaghetti, over 90 g/100 g, confirming cassava as a carbohydrate-rich food [14], followed by cassava:sorghum pasta (C50:S50). Pasta samples containing durum wheat semolina (S50:DS50 and C50:DS50) showed the lowest values of starch, 73 g/100 g, whereas the remaining formulations showed 81 g/100 g on average. Resistant starch content was found in all pasta samples below the limit of 2% reported by the method; however, the highest (0.525 g/100 g) and the lowest (0.170 g/100 g) contents were observed when cassava flour was combined with 50% durum wheat and 50% sorghum whole meal flours, respectively. Durum wheat semolina pasta (DS100) showed high amylose content without significant differences compared to spaghetti obtained from mixtures with 50% cassava flour (C50:DS50, C50:S50, and C50:DW50) (Table 2), whereas the 100% cassava spaghetti exhibited the lowest content. However, as observed by Masato et al. [28] in durum wheat, the amylose content in all the dried pasta samples slightly increased but the differences were not significant with respect to flours. Foods rich in amylose are associated with a drop in blood glucose levels and more gradual emptying of the human gastrointestinal tract versus those with low levels of amylose [22]; hence, cassava’s healthy potential could be increased by its association with other high amylose content materials such sorghum or durum wheat flours. Results from the total dietary fiber analysis revealed the lowest values in pasta formulations containing refined semolina (DS100, C50:DS50, and S50:DS50) due to the removal of the outer kernel layers, which are rich in fiber. Conversely, spaghetti from cassava flour combined with durum wheat whole meal flour (C50:DW50), in which the outer layers of the kernels are present, showed TDF values of up to 10 g/100 g, almost 3 percentage points more than 100% cassava spaghetti (C100). As expected, and due to refinement processing, pasta formulations containing semolina and cassava flour, DS100, C100, and C50:DS50, presented the lowest total antioxidant capacities (Table 2), whereas higher levels were reported in pasta formulations containing whole meal flours. These results are mostly reflected in the greatest concentration of antioxidant compounds in the kernel external layers [29], polyphenols and phenolic acids, accounting for the most representative part, especially in sorghum. As stated in the Introduction section, pasta, made with sorghum and durum whole meal flours, could be a valuable vehicle of these compounds in contributing to healthy status maintenance. Regarding ash content, the C50:DW50 pasta sample reported the highest value (Table 2), followed by mixtures with 50% of sorghum whole meal flour; pasta formulations that included refined flours (DS100, C100, and C50:DS50) did not reach 0.9 g/100 g ash content, which is the limit imposed by the FAO/WHO Codex Alimentarius of current regulations for semolina. This is ascribed to the greater mineral amounts in cereal external layers [27]. With minerals being important for healthy human body functioning, the increase in their content in pasta formulations containing cassava also contributes to the valorization of this species. Moreover, with food-grade sorghum characterized by high Mg, Fe, and Zn content, high K:Na ratio and low Ca:P ratio compared to other crops [30], the use of blends containing sorghum in pasta product could contribute to an increase in nutritional potential.

### 2.3. Cooking Quality Parameters and Sensory Evaluation of Pasta Samples

**Figure 1:**
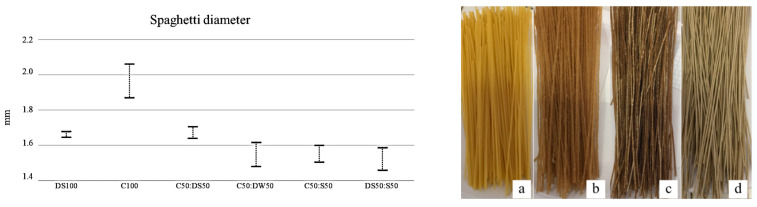
On the left, the range of the variability of dried spaghetti diameter (mm); the upper and the lower edges of the bars indicate the maximum and minimum values in each pasta formulation. On the right, dried spaghetti from: DS100 (**a**), C100 (**b**), C50:S50 (**c**), and DS50:S50 (**d**). C: cassava; S: sorghum; DS: durum wheat semolina; DW: durum wheat whole meal.

**Figure 2:**
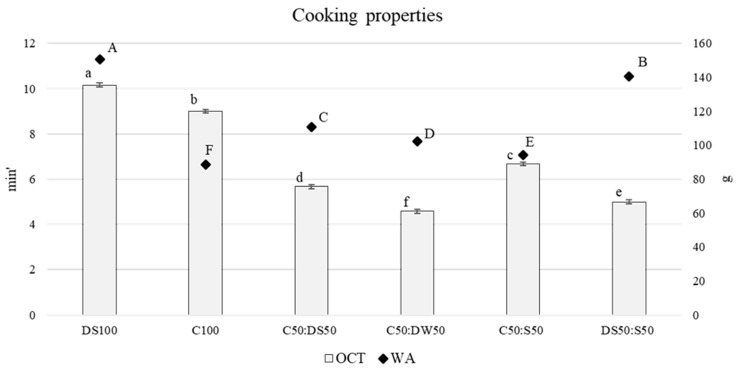
Cooking properties of the different pasta formulations. OCT: optimal cooking time (min); WA: water absorption (g; ♦); C: cassava; S: sorghum; DS: durum wheat semolina; DW: durum wheat whole meal. Different letters (uppercase for WA and lowercase for OCT) indicate differences determined using Duncan’s test (*p* ≤ 0.05). Results are expressed as ± standard deviation for three replications.

The variation in spaghetti diameter (Figure 1) was low in semolina pasta due to the homogeneous distribution of the gluten matrix along the entire spaghetti length, whereas large variability was observed in other pasta samples. Indeed, the use of gluten-free materials or the incorporation of bran weakens the gluten network, causing a rough surface responsible for the variation in spaghetti diameter during the drying phase [31]. Semolina pasta (DS100) showed the highest optimal cooking time (OCT), almost double with respect to the other pasta formulations containing durum wheat, except for 100% cassava spaghetti (C100) and C50:S50 (Figure 2). C100 pasta showed OCT longer than the other formulation, likely because of the lower starch content due to the partial replacement of cassava flour [32]. The replacement of part of the semolina with cassava or sorghum in C50:DS50 and DS50:S50 samples significantly decreased the OCT, likely because of the lower homogeneity of gluten distribution that makes water access easier, as also confirmed by the water absorption (WA) data (Figure 2). Moreover, the shorter cooking time in the cassava:durum wheat pasta formulation, with respect to C100 pasta, could be attributed to its higher amylose content, i.e., lower amylopectin content, which is reflected in a more rapid gelatinization of the starch granules [33]. Indeed, cassava starch is characterized by a high ratio of amylopectin/amylose [34]. The high amylopectin content of cassava starch also increased the OCT of sorghum:cassava pasta with respect to sorghum:semolina pasta; this could be ascribed to the characteristics of sorghum starch, which requires a longer amount of time than wheat to reach complete gelatinization [35]. With sorghum starch slowly digested [36], the inclusion of flours to the blends for pasta making could contribute to a reduction in the glycemic index of cassava, which is known to be high [22].

Water absorption, i.e., the weight increase from dry to cooked pasta at the optimum cooking time, was significantly higher in semolina pasta (DS100), followed by spaghetti, which included percentages of semolina or whole meal durum wheat flour, DS50:S50, C50:DS50, C50:DW50, as reported in Figure 2. Water absorption of the remaining pasta formulations showed lower values due to the characteristics of the materials used for pasta making. In particular, semolina pasta absorbed more cooking water than gluten-free cassava pasta since gluten is responsible for higher water absorption and longer cooking time [37]. This is further demonstrated by the higher amount of water absorption in pasta formulations containing durum wheat (Figure 2).

**Figure 3:**
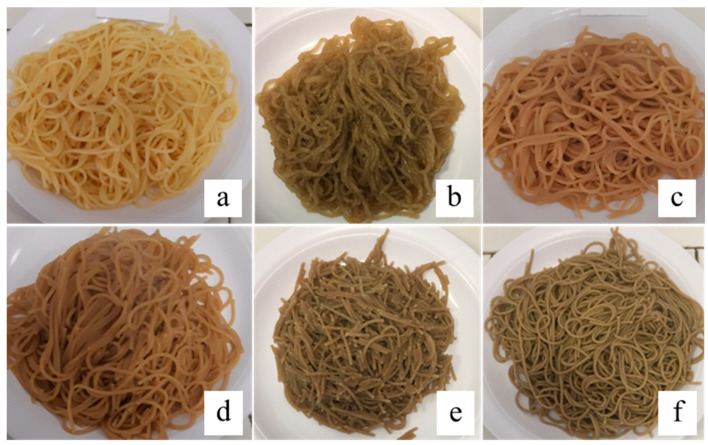
Cooked spaghetti from DS100 (**a**), C100 (**b**), C50:DS50 (**c**), C50:DW50 (**d**), C50:S50 (**e**), and DS50:S50 (**f**) pasta formulations. C: cassava; S: sorghum; DS: durum wheat semolina; DW: durum whole meal wheat.

**Figure 4:**
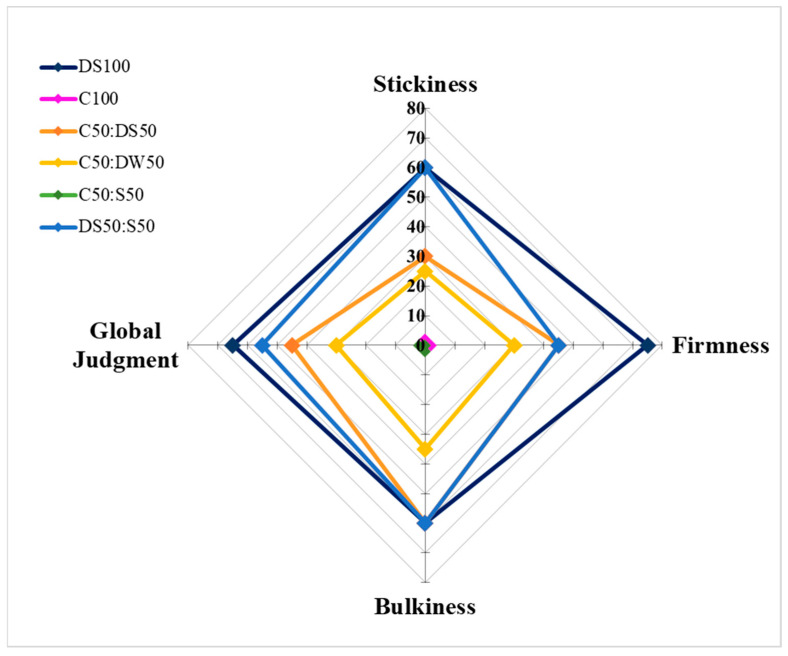
Radar chart of sensory analysis of the six pasta samples. C: cassava; S: sorghum; DS: durum wheat semolina; DW: durum wheat whole meal. Results are expressed as mean for five replications. Firmness: absent (≤20), rare (>20 and ≤40), sufficient (>40 and ≤60), good (>60 and ≤80), very good (>80 and ≤100); bulkiness and stickiness: very high (≤20), high (>20 and ≤40), rare (>40 and ≤60), almost absent (>60 and ≤80), absent (>80 and ≤100); global judgment: scarce (<55), sufficient (≥55 and <65), good (≥65 and <75), very good (≥75).

The sensory analysis, focused on texture quality traits, was carried out on pasta samples cooked at the optimum cooking time (Figure 3). The highest scores for firmness (75), bulkiness (60), stickiness (60), and overall judgment (65) were revealed in semolina pasta (DS100); followed by spaghetti, which included semolina in their formulation; DS50:S50; and C50:DS50 (Figure 4). In particular, spaghetti made from 50% semolina and 50% sorghum flour (DS50:S50) showed the same indices of stickiness and bulkiness observed for semolina pasta, whereas firmness and global judgment (GJ) were lower but in the range considered sufficient (≥55 and ≤65) for the semolina pasta sensorial quality standard [38,39]. As expected, gluten-free spaghetti with cassava and sorghum C100 and C50:S50 obtained the worst sensorial judgment (Figure 4), with C50:S50 resulting in a completed fragmented spaghetti over cooking (Figure 3e) as a consequence of the absence of a gluten network and of the lack of additives in the recipes along with the use of the traditional semolina pasta-making process. Pasta samples of cassava flour blended with durum wheat whole meal (C50:DW50) showed high values of stickiness (25) and bulkiness (35) that led the global judgment (30) to be below the acceptability limit referred to the durum wheat pasta. However, it should be noted that the cooking quality linked to the spaghetti texture, refer to semolina pasta and reflect the Italian consumers’ preferences. The use of different raw materials is expected to affect sensory attributes of pasta; however, foreign consumers accustomed to consuming sorghum and cassava products could consider these pasta products palatable. Further investigations on the hedonistic aspects and setup of pasta-making are needed to make these products more appreciated on the market.

## 3. Materials and Methods

### 3.1. Plant Material

Cassava (*Manihot esculenta* Crantz) flour was purchased from an ethnic market in Rome (Italy), food-grade sorghum hybrid (AG4E44 line) was kindly provided by Padana Sementi Elette (Tombolo, PD, Italy) and cultivated in Rome (Italy) at the CREA-IT experimental fields, whereas durum wheat, from a mix of thirty cultivars, was obtained from the durum wheat Italian National Trials grown in Catania (Italy). The three raw materials were used to produce six different formulations of dry pasta (Table 3).

### 3.2. Milling and Pasta-Making Process

Cassava flour, sorghum, and durum wheat kernels were micronized with the Pulverisette apparatus (Fritch, Idar-Oberstein, Germany), with a 0.7 mm sieve. Durum wheat kernels were milled using a pilot plant (Buhler MLU 202, Uzwill, Switzerland) to obtain semolina.

A laboratory mill (Cyclotec, FOSS, Hillerod, Denmark) with a 0.5 or 1.0 mm sieve according to chemical analyses official methods was used.

Different percentages of cassava, sorghum, durum whole wheat flours, and semolina were applied to produce six types of pasta in spaghetti shape, as reported in Table 3. The 100% durum wheat semolina was considered as the reference sample. The 100% sorghum pasta could not be produced due to the lack of gluten coupled with the starch characteristics (high gelatinization temperature) that prevented spaghetti from been extruded.

The different flour formulations were mixed with tap water to obtain 37% dough moisture and spaghetti was obtained by using an experimental press (NAMAD, Rome, Italy) with a capacity up to 20 kg/h, equipped with a Teflon-coated extruder consisting of 164 holes, 1.80 mm diameter, at the following conditions: 1 bar chamber vacuum; 15 min kneading; 50 °C die temperature; 42 rpm auger extrusion speed. An experimental dryer (AFREM, Lyon, France) was used, applying a low temperature drying program (Tmax = 58 °C for 18 h) and a linear decrease in the relative humidity into the drier chamber from 85% to 70% during the entire drying process [40]. The final pasta moisture content was ≤ 12.5%. Spaghetti samples were stored at room temperature until analyses. For each pasta sample, 3 spaghetti strands were chosen at random, and the mean diameter was measured using a caliper.

### 3.3. Chemical Characterization and Total Antioxidant Capacity of Dry Pasta

Chemical composition and total antioxidant capacity were assessed both on raw materials and dry pasta. All analytical determinations were performed in triplicate and data were expressed as dry weight by measuring moisture using the Sartorius M40 thermobalance (Goettingen, Germany) at 120 °C just before the chemical analyses.

Protein content was determined according to the ICC 105-2 method [41]. Total and resistant starch (TS and RS) were measured using the enzymatic method with the Megazyme (Bray, Ireland) kits K-TSTA and KRSTAR according to AOAC methods 996.11 [42] and 2002.02 [43], respectively. Amylose content was determined using the Megazyme Amylose/Amylopectin assay kit K-AMYL. Total dietary fiber (TDF) content was measured according to official method 991.43 [44] using the enzymatic kit Bioquant (Merck, Darmstadt, Germany). For ash content determination, official method 08-01.01 [45] was applied. Total antioxidant capacity (TAC) was determined according to Martini et al. [46]. In detail, a solution made of 7 mM ABTS radical and 2.45 mM potassium persulfate was diluted with 50% ethanol to reach an absorbance of 0.7 OD at 734 nm. The sample (100 mg), diluted 1/10 *w*/*w* with cellulose powder, was immersed in the radical solution, incubated in an orbital shaker at 25 °C for 50 min, and then centrifuged at 10,500 rpm. TAC was expressed as mmol of Trolox-equivalent antioxidant capacity per kilogram of dry matter (mmol of TEAC/kg dm) using a Trolox dose–response curve.

### 3.4. Cooking Quality and Sensory Evaluation of Cooked Pasta

Pasta samples were cooked according to the AACC method 66–50.01 [47] by adding one hundred grams of dried spaghetti to 1 L of boiling tap water without salt. Optimum cooking time (OCT) was determined according to D’Egidio et al. [38] and corresponds to the disappearance of the starchy central core of spaghetti when squeezed between two glasses. Water absorption (WA) was calculated as the weight increase in pasta at the OCT and determined as: WA = [(w − w0)/w0] × 100, where w and w0 were the weight of cooked and raw pasta, respectively. Cooking loss (CL), expressed as grams of matter loss/100 g in raw pasta, was evaluated by weighing the residues of solids lost to the cooking water after drying overnight at 105 °C.

In detail, for sensorial judgment, three textural characteristics were evaluated using a score ranging from 10 to 100: (i) firmness, which indicates the resistance to chewing by the teeth; (ii) stickiness, which consists of the material adhering to the cooked pasta surface; (iii) bulkiness, which is the degree of jamming among the spaghetti strands. The global judgment (GJ) was the arithmetic mean of the three textural components [40]. The sensorial judgment, based on three textural characteristics of cooked pasta, was evaluated by a panel of five trained technician assessors, four female and one male, aged from 40 to 62, as reported by Gazza et al. [40].

### 3.5. Statistical Analysis

Results were expressed as mean ± standard deviation. One-way analysis of variance was performed with MSTATC software (Michigan State University, East Lansing, MI, USA), followed by the Duncan multiple range test for post-hoc comparison of means, applied to assess significant differences (*p* ≤ 0.05) for each considered parameter.

## 4. Conclusions

The exploration of novel raw materials for the production of the most typical Italian food, dry pasta, was conducted to address the demand for typical foods from African migrants. The addition of sorghum or durum wheat to cassava resulted in an increase in nutritional potential in terms of protein content and total antioxidant capacity of cassava, of which it is deficient. Similarly, the textural and cooking properties of cassava pasta were improved by enrichment with durum wheat, which is the most suitable for the production of dry pasta. The incorporation of durum wheat semolina determined the same meliorative effect on the overall sorghum cooking quality parameters, confirmed by the sufficient global judgment score reached using sorghum:semolina pasta. Overall, the addition of 50% semolina both to sorghum and cassava flours resulted in the best compromise in terms of nutritional and technological and sensorial aspects.

All spaghetti samples indicated the need for technological adjustments to pasta processing, such as steaming treatment of sorghum flour in order to increase the starch gelatinization to improve the spaghetti’s texture. Moreover, the identification of the suitable amount of wheat bran to cassava flour could be a strategy to increase the sensorial judgment score and, in particular, firmness.

## Figures and Tables

**Table 1:** Chemical composition and total antioxidant capacity of cassava, sorghum and durum wheat whole flours, and semolina.

	Protein	TS	RS	Amylose	TDF	TAC	Ash
Flour	(g/100 g)	(g/100 g)	(g/100 g)	(g/100 g)	(g/100 g)	(mmol TEAC/kg)	(g/100 g)
CF	2.25 ± 0.07 ^c^	86. 9 ± 0.9 ^a^	0.30 ± 0.01 ^b^	20 ± 4 ^c^	7.5 ± 0.2 ^c^	18.9 ± 0.4 ^c^	0.77 ± 0.01 ^d^
SF	9.4 ± 0.2 ^b^	77 ± 1 ^b^	0.37 ± 0.01 ^a^	23.3 ± 0.1 ^bc^	12.2 ± 0.1 ^b^	34.3 ± 0.2 ^b^	1.888 ± 0.003 ^b^
DWF	14.936 ± 0.005 ^a^	64 ± 4 ^c^	0.31 ± 0.01 ^b^	25 ± 2 ^b^	12.90 ± 0.09 ^a^	57 ± 1 ^a^	1.974 ± 0.009 ^a^
DS	14.07 ± 0.03 ^b^	79 ± 2 ^b^	0.262 ± 0.005 ^c^	29.3 ± 0.1 ^a^	4.6 ± 0.3 ^d^	35.7 ± 0.1 ^b^	0.860 ± 0.006 ^c^

Results are reported as dry weight and expressed as mean ± standard deviation for three replications. Within the same column, values with different letters indicate significant differences determined using Duncan’s test (*p* ≤ 0.05). TS: total starch; RS: resistant starch; TDF: total dietary fiber; TAC: total antioxidant capacity; TEAC: Trolox-equivalent antioxidant capacity; CF: cassava flour; SF: sorghum whole meal flour; DWF: durum wheat whole meal flour; DS: durum wheat semolina.

**Table 2:** Chemical traits and total antioxidant capacity of dried pasta samples.

	Proteins	TS	RS	Amylose	TDF	TAC	Ash
Pasta	(g/100 g)	(g/100 g)	(g/100 g)	%	(g/100 g)	(mmol TEAC/kg)	(g/100 g)
DS100	13.86 ±0.04 ^a^	78.9 ± 0.5 ^c^	0.310 ± 0.007 ^c^	28.9 ± 0.9 ^a^	4.34 ± 0.06 ^f^	29.9 ± 0.5 ^d^	0.85 ± 0.01 ^e^
C100	2.10 ± 0.07 ^e^	91.7 ± 0.2 ^a^	0.27 ± 0.02 ^d^	22.1 ± 0.3 ^c^	8.32 ± 0.05 ^c^	21.2 ± 0.4 ^e^	0.83 ± 0.01 ^f^
C50:DS50	5.97 ± 0.07 ^c^	73 ± 2 ^d^	0.417 ± 0.005 ^b^	29.4 ± 0.6 ^a^	5.04 ± 0.07 ^e^	31.1 ± 0.2 ^d^	0.915 ± 0.004 ^d^
C50:DW50	9.81 ± 0.05 ^b^	79.8 ± 0.1 ^c^	0.525 ± 0.001 ^a^	29.0 ± 0.8 ^a^	11.0 ± 0.1 ^a^	35.1 ± 0.5 ^b^	1.535 ± 0.002 ^a^
C50:S50	5.3 ± 0.2 ^d^	84 ± 2 ^b^	0.170 ± 0.003 ^e^	29.2 ± 0.4 ^a^	8.83 ± 0.01 ^b^	33 ± 1 ^c^	1.26 ± 0.05 ^c^
DS50:S50	9.9 ± 0.1 ^b^	73 ± 1 ^d^	0.510 ± 0.005 ^a^	25 ± 1 ^b^	5.4 ± 0.1 ^d^	39.9 ± 0.5 ^a^	1.372 ± 0.006 ^b^

Results are reported as dry weight and expressed as mean ± standard deviation for three replications. Within the same column, values with different letters indicate significant differences determined using Duncan’s test (*p* ≤ 0.05). TS: total starch; RS: resistant starch; TDF: total dietary fiber; TAC: total antioxidant capacity; TEAC: Trolox-equivalent antioxidant capacity; C: cassava; S: sorghum; DS: durum wheat semolina; DW: durum wheat whole meal.

**Table 3:** Pasta formulations from cassava, durum wheat, and sorghum flours.

	Pasta Formulations (%)					
Flours	DS100	C100	C50:DS50	C50:DW50	C50:S50	DS50:S50
Cassava	-	100	50	50	50	-
Sorghum whole meal	-	-	-	-	50	50
Durum wheat semolina	100	-	50	-	-	50
Durum wheat whole meal	-	-	-	50	-	-

C: cassava; DS: durum wheat semolina; DW: durum wheat whole meal; S: sorghum whole meal.

## Data Availability

Data are contained within the article.
